# Peach LAZY1 and DRO1 protein-protein interactions and co-expression with PRAF/RLD family support conserved gravity-related protein interactions across plants

**DOI:** 10.17912/micropub.biology.000995

**Published:** 2024-01-10

**Authors:** Jessica M. Waite, Courtney A. Hollender, Jon R. Eilers, Erik Burchard, Chris Dardick

**Affiliations:** 1 USDA ARS Tree Fruit Research Laboratory, Wenatchee, WA; 2 Department of Horticulture, Michigan State University, East Lansing, MI; 3 USDA ARS Appalachian Fruit Research Station, Kearneysville, WV

## Abstract

IGT/LAZY proteins play a central role in determining gravitropic set point angle and orientation of lateral organs across plant species. Recent work in model systems has demonstrated that interactions between IGT/LAZY proteins and BREVIS RADIX (BRX)-domain containing proteins, such as PH, RCC1, AND FYVE/RCC1-LIKE DOMAIN (PRAF/RLD), and BREVIS RADIX LIKE (BRXL) family members, are mechanistically important for setting gravitropic set point angle. Here, we identified peach PRAF/RLD proteins as interactors of the peach IGT/LAZY proteins PpeLAZY1 and DEEPER ROOTING 1 (PpeDRO1) from a yeast-two-hybrid screen. We also show that the BRX domains of these interacting proteins have high sequence similarity with PRAF/RLD and BRX family proteins from rice and Arabidopsis. Further, PpeLAZY1 and the peach PRAF/RLD interactors are all expressed at relatively high levels in leaf, meristem, and shoot tip tissues. Together, this evidence supports the importance and conservation of IGT/LAZY-BRX-domain interactions, which underlie setting gravitropic set point angle across angiosperms.

**Figure 1. Peach PRAF1/RLD protein-protein interaction and co-expression with PpeLAZY1 and PpeDRO1:**
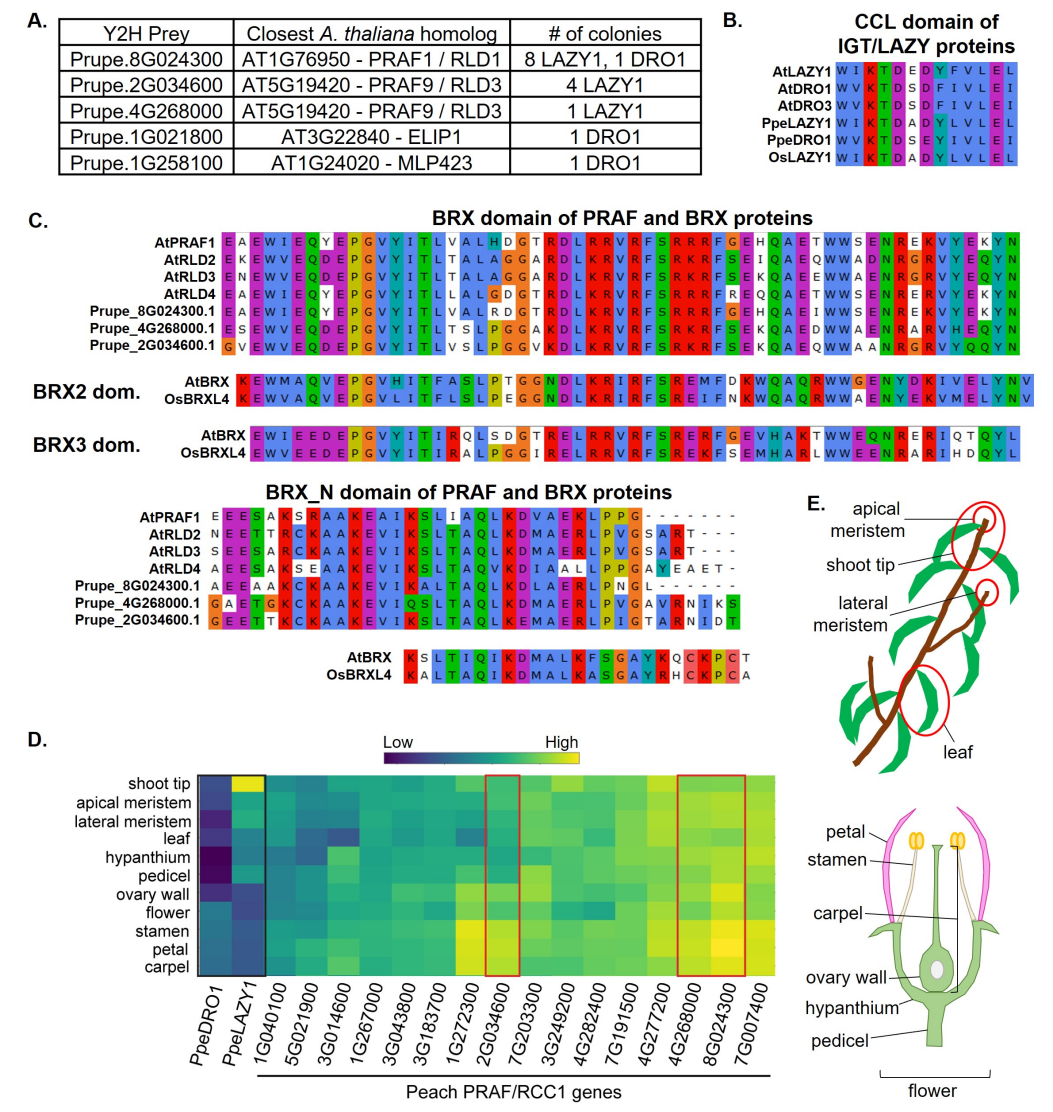
(A) Results from a yeast-two-hybrid screen, using the coding sequence (CDS) of PpeLAZY1 or PpeDRO1 as bait proteins. Identities of yeast-two-hybrid prey interactors (left column) after sequencing, and their closest Arabidopsis homologs (center column) are reported, along with the number of yeast colonies (indicating positive interactions) from which each interactor was identified. (B) Alignment of the CCL domain in a selection of Arabidopsis, peach, and rice IGT/LAZY genes, shows a high degree of conservation across species. IGT/LAZY proteins included here encompass all family members for which PRAF/RLD or BRX family proteins have been identified as interactors, including this study (Li et al., 2019; Furutani et al., 2020; Che et al., 2023). Arabidopsis, peach, and rice have four, four, and seven additional IGT/LAZY family members, respectively (Waite and Dardick, 2021). (C) A high degree of similarity is seen in protein alignments of the BRX, BRX2, BRX3, and BRX_N domains from PRAF/RLD and BRX proteins from Arabidopsis, peach, and rice genomes. Protein domains are defined by InterPro (BRX: IPR013591, BRX_N: IPR027988) and Briggs et al. (Briggs et al., 2006). (D and E) Gene expression heatmap of PpeLAZY1, PpeDRO1, and peach PRAF/RLD genes across aerial tissues (leaf, meristem, shoot tip, and floral tissues, represented pictorially in E) shows co-expression in some tissues, particularly in leaf and meristem tissues for PpeLAZY1 and floral tissues for PpeDRO1. Scale is based on normalized, log transformed counts. Black rectangle indicates PpeLAZY1 and PpeDRO1, red rectangles indicate peach PRAF proteins identified in the yeast-two-hybrid screen as interactors.

## Description

Gravitropism, or the capacity of plants to sense gravity and alter their growth and orientation, is central to plant development. The direction and angle at which lateral root or shoot branches grow with respect to gravity is often referred to as their gravitropic set-point angle. The gravitropic set point angle of lateral shoots and roots is important for shaping plant architecture (Digby and Firn, 1995) . It also influences plants’ access to nutrients, water, sunlight, and beneficial microbial communities. Gravitropic set point angle can be influenced by environmental factors such as light and nutrients (Digby and Firn, 2002; Roychoudhry et al., 2017; Waite and Dardick, 2018) , endogenous physiological components such as changes in auxin (Roychoudhry et al., 2013) , and genetic determinants, including the *IGT/LAZY* family of genes, which includes several *LAZY* , *DEEPER ROOTING (DRO)* and *LAZY1-* LIKE genes as well as *TILLER ANGLE CONTROL 1 (TAC1)* (Waite and Dardick, 2021; Kawamoto and Morita, 2022) .

*IGT/LAZY* genes play a key role in determining gravitropic set point angle. The *lazy1* mutant shoot architecture trait was first described in the 1930’s in maize and rice having horizontal tiller growth (Van Overbeek, 1936; Jones and Adair, 1938) . Since then, much research has worked towards elucidating the mechanisms underlying IGT/LAZY protein influence on shoot and root gravitropic set point angle, primarily using model species (Nakamura et al., 2019) . Further, these genes have been identified and characterized in an increasing number of ornamental and crop species, including *Prunus persica* (peach), *Malus domestica* (apple), *Lotus japonicus* (Lotus), *Brassica napa* , *Populus* *trichocarpa* (poplar), *Betula pendula* (silver birch), and *Triticum sp.* (wheat) (Dardick et al., 2013; Li et al., 2017; Salojarvi et al., 2017; Wang et al., 2018; Ashraf et al., 2019; Chen et al., 2020; Fladung, 2021) . Until recently, protein-protein interactions specific to IGT/LAZY proteins were unknown, but work in Arabidopsis and rice has uncovered interactions between the C-terminal domains of LAZY and DRO proteins and the BRX domains of both PRAF/RLD and BRX-Like proteins (Li et al., 2019; Furutani et al., 2020; Che et al., 2023) .

To determine protein-protein interactions specific to the peach IGT/LAZY proteins PpeLAZY1 (Prupe.1G222800) and PpeDRO1 (Prupe.3G038300), we constructed a yeast-two-hybrid library of peach proteins and bait vectors expressing coding sequences from PpeLAZY1 and PpeTAC1. Each bait was mated with the library and resulting yeast were plated on appropriate selection. Putative positive colonies were re-plated on more stringent selective media. DNA from the subsequent colonies was sequenced to determine the genes coding for the interacting proteins. We identified 13 successful interactions with the PpeLAZY1 bait, and 3 with PpeDRO1. After sequencing, 5 distinct gene sequences were identified, 3 of which accounted for 14 of the 16 total interactions and all belong to the PRAF/RLD family of proteins ( Fig. 1A ). The PRAF/RLD proteins are characterized by containing Pleckstrin Homology (PH), Regulator of chromosome condensation (RCC1), and FYVE zinc finger domains, and belong to a larger family of FYVE domain-containing proteins (Wywial and Singh, 2010) . They also contain a Brevis Radix (BRX) protein-protein interaction domain, similar to BRX and BRX-Like proteins (Briggs et al., 2006) .

In addition to the BRX domain-containing proteins, two other interacting proteins were identified from the PpeDRO1 screen: peach proteins most closely related to the Arabidopsis EARLY LIGHT-INDUCIBLE PROTEIN 1 (ELIP1) and MAJOR LATEX PROTEIN 423 (MLP423). ELIP1 is induced strongly by high light and can be co-isolated with the Light Harvesting Complex II involved in photosynthesis (Heddad et al., 2006) . Interestingly, both *ELIP1* and *DRO1* expression is regulated by the light signaling transcription factor, HY5 (Harari-Steinberg et al., 2001; Yang et al., 2020) . While a mechanism is unknown, *DRO1* expression is influenced by light and *IGT/LAZY* triple and quadruple mutants lose responses to light (Waite and Dardick, 2020) . This could suggest a role for ELIP1 in this connection. MLP423 is associated with multiple types of stress, and is a potential target of an F-box protein involved in leaf polarity and shoot apical meristem organization (Abercrombie et al., 2008; Ascencio-Ibanez et al., 2008; Kosmacz et al., 2019) . There are fewer focused studies on this protein, however, and the potential connection with DRO1 is less clear.

To further investigate the peach interactors belonging to the *PRAF/RLD* gene family, we analyzed the specific sequences inserted into the yeast-two-hybrid prey library vectors. We found that for all PRAF/RLD interactors, the sequence fragments present in the prey vectors contained a BRX or BRX_N domain. Recent work in Arabidopsis demonstrated that the BRX domain of AtPRAF/RLD proteins interacts with the C-terminal domain (CCL) of AtDRO1, and that this interaction is central to polar localization of IGT/LAZY proteins at the plasma membrane and resulting gravitropic growth (Furutani et al., 2020; Nishimura et al., 2023) . Our yeast-two-hybrid results suggest that this interaction is conserved in peach. CCL domain alignments of peach IGT/LAZY proteins and Arabidopsis and rice homologs that have been previously shown to interact with BRX-domain-containing proteins show high similarity ( Fig. 1B ). Next, we aligned the Peach BRX domain sequences from our interactors with the same domains from Arabidopsis PRAF/RLD proteins to visualize amino acid differences between the various proteins and found these domains to have very high sequence similarity, demonstrating conservation across dicotyledonous plants ( Fig. 1C ). These sequences also aligned well with BRX and BRX_N domains from Arabidopsis and rice BRX and BRXL proteins shown to interact with IGT/LAZY proteins ( Fig. 1C ). Together this offers further support for the BRX domain being a key domain for interaction with the IGT/LAZY proteins across protein families and plant species.

To determine whether the identified interactors are expressed in a similar spatiotemporal context as the *IGT/LAZY* genes in peach we performed tissue-specific analyses of published and unpublished RNA seq gene expression datasets (Table S1). Transcript counts derived from a variety of peach shoot and flower tissues were collectively normalized to enable for transcriptomic comparison. We analyzed gene expression profiles of peach *IGT/LAZY* and *PRAF/RLD* gene family members and found that *PpeLAZY1* and the *PRAF/RLD* interactors all had relatively high expression in the shoot tip, shoot meristems, and leaves, supporting co-expression in these tissues. In floral tissues, these *PRAF/RLD* genes were also highly expressed, however *PpeLAZY1* was downregulated. *PpeDRO1* showed little expression in shoot and floral tissues. This is likely due to *PpeDRO1* expression being largely restricted to root tissues (Guseman et al., 2017) , however we were unable to identify or obtain any root tissue expression data for this experiment. Taken together, this suggests that in non-floral shoot organs, PpeLAZY1 and peach PRAF/RLD proteins are expressed in similar tissues and are likely to form functional interactions.

## Methods

Y2H library construction

Total RNA was extracted from shoots of mature field grown peach “Encore” trees using Trizol reagent according to manufacturer protocols (ThermoFisher Scientific). A total of 2mg of total RNA was subjected to mRNA enrichment using an Oligotex kit to yield 20ug of mRNA (Qiagen). 8ug of mRNA was used to make the yeast two-hybrid cDNA library using the HybriZAP®-2.1 two-hybrid cDNA gigapack cloning kit and HybriZAP®-2.1 two-hybrid cDNA synthesis kit (Agilent Technologies) with the following modifications: (1) replaced the Stratascript reverse transcriptase (RT) with Invitrogen Superscript III RT (ThermoFisher Scientific); (2) Amersham GFX columns were used for all cDNA purification steps and cDNA size selection columns were used to remove cDNAs <600bp in place of drip columns per HybriZAP instructions. To make the library, 150ng of size selected cDNA was used for ligations and 1uL of cDNA was used for phage packaging a total of five times. This yielded a primary library size of 2.5 X 10e6 cDNA clones. The resulting library was amplified and stored at -80 ^o^ C prior to excising phagemid. For library screening, amplified phagemid was purified using the Qiagen Maxi prep kit (Qiagen) and co-transformed with linearized pGADT7 vector (Activation Domain/prey vector) into the yeast strain Y187 via the Yeast transformation kit, Frozen-EZ yeast transformation II (Zymo Research).

Yeast-two-hybrid Bait construct cloning and transformation

To generate the yeast-two-hybrid Binding Domain (BD/bait) vectors with the pXDGAT CY86 backbone (Ding et al., 2004) , the full length peach *LAZY1* and *DRO1* CDS sequences were amplified by RT-PCR from peach RNA using the following primers: FL-LAZY-for-YTH-F 5’-CAC CAT GAA GTT ACT AGG TTG GAT GCA TCG-3’ and FL-LAZY1-for-YTH-R 5’-CTT CAT TTC ACC TTT CAC AGC TCC; PpDRO1-CDS-F 5’ATG AAG CTT TTT GGT TGG ATG-3’ and PpDRO1-CDS-R 5’-TCA AAT TTC TAG GAC TAT ATA TTC AGA ATC TGT-3’. The resulting amplicons were TA-cloned into the pENTR/D-TOPO vector (Invitrogen, ThermoFisher) and then cloned into pXDGAT CY86 via LR-reaction to produce PpeLAZY1-BD and PpeDRO1-BD (Ding et al., 2004) . The yeast-two-hybrid pXDGAT BD vectors were transformed into the yeast strain AH109 according to the Clontech Yeast Protocols Handbook PT3024-1 (Clontech, now Takara Bio USA).

Yeast-two-hybrid mating and selection

Yeast containing BD (bait, PpeLAZY1 and PpeDRO1) and AD (prey library) vectors were mated and plated according to the Clontech Yeast Protocols Handbook PT3024-1 (Clontech, now Takara Bio USA). Briefly, cultures of AH109 cells containing pXDGAT-PpeLAZY1-BD and pXDGAT-PpeDRO1-BD (bait vectors) were grown overnight, spun down, and resuspended in YPD to a concentration of ~2x10e8 cells. Library/prey aliquots equal to ~1x10e8 cells were added to the cultures, and yeast were incubated at 30 ^o^ C with shaking for 3-4 hours. Liquid was removed via vacuum, such that mated yeast remained on a filter membrane, which was then placed yeast-side-up on YPD plates (pH 4.5) and incubated overnight at 30 ^o^ C. Yeast were then washed off the membrane and plated on selection plates (SD -His, -Leu, -Trp +3AT). Some yeast was reserved for serial dilutions to be plated on less stringent media to determine the number of clones screened. Colonies that formed on screening plates were picked and re-streaked on SD -Trp and SD -His, -Leu, -Trp plates. Yeast from these was lysed, DNA-extracted, sequence amplified with PCR, and subjected to Sanger sequencing. Sequences were screened via BLAST against the peach genome (v2.0) to determine the identity of the interactors (Verde et al., 2017) .

Protein domain alignments

Protein sequences for peach proteins were obtained from the Prunus persica v2.0 genome from the Genome Database for Rosaceae (Verde et al., 2017; Jung et al., 2019) , for Arabidopsis from the TAIR10 genome from The Arabidopsis Information Resource (Lamesch et al., 2012) , and for rice from the Rice Genome Annotation project 7 (Kawahara et al., 2013) . Plant Tribes 2 (Wafula et al., 2023) was used to identify orthogroups and further validate the relationships of the peach, rice, and Arabidopsis homologs. The resulting protein sequences were then loaded into UGENE (Okonechnikov et al., 2012) , BRX and CCL domains were identified and extracted, and realigned using the MUSCLE algorithm and default values.

Gene expression

Tissue was collected in liquid nitrogen from dormant bud sticks allowed to grow out in the greenhouse or from field grown trees. Tissues were hand dissected and ground to powder in liquid nitrogen using a mortar and pestle, or lyophilized and ground using metal beads and a homogenizer. RNA was extracted using either a Norgen Plant/Fungi Total RNA Purification Kit (Norgen Biotek, Thorold, ON, Canada) or an Omega SQ Total RNA kit with 2% polyvinylpyrrolidone added to the red cell lysis buffer (Omega Bio-tek, Norcross, GA). RNA was sent for sequencing using Illumina Hi-Seq.

RNA sequences from multiple datasets were analyzed for this study to cover an array of tissue types. Differential gene expression from some of these datasets has been published previously: data from shoot tips were included in (Hollender et al., 2018) ; hypanthium and ovary wall in (Li et al., 2022) ; petal, carpel, and stamen in (Zhu et al., 2020) . Table S1 gives the NCBI accession numbers for all datasets. Raw sequencing reads from all tissues were re-mapped to the Prunus persica v2.0 genome (Verde et al., 2017) using STAR v2.7.10a transcript alignment software with default parameters (Dobin et al., 2013) . Counts were performed using featureCounts v2.0.3 from the Subread software package (Liao et al., 2014) . Counts from all tissues were combined into a single counts table and normalized using TMM (Trimmed Mean of M-values) to calculate effective library sizes using the R (v4.1.0, https://www.r-project.org) package edgeR’s (v3.34.1) calcNormFactors function (Robinson et al., 2010) . Normalized counts were converted to log counts per million (CPM), mean CPM was calculated for each tissue type for each of the genes of interest, and mean values were used as input to the R package heatmaply, v1.4.2.
